# Peptide/Protein Functionalization and Macrocyclization via Alkyne Umpolung with Hypervalent Iodine Reagents

**DOI:** 10.1021/acs.accounts.5c00451

**Published:** 2025-08-29

**Authors:** Xing-Yu Liu, Jerome Waser

**Affiliations:** Laboratory of Catalysis and Organic Synthesis, 27218 Ecole Polytechnique Fédérale de Lausanne, EPFL SB ISIC LCSO, BCH 4306, 1015 Lausanne, Switzerland

## Abstract

Alkynes are one of the most fundamental functional groups in organic synthesis due to the versatile chemistry of the triple bond, their unique rigid structure, and their use in bioconjugation. The introduction of alkynes onto organic molecules traditionally relies on nucleophilic activation, often requiring strong bases or metal catalysts. These conditions, however, restrict applications involving biomolecules such as peptides and proteins due to functional group incompatibility. To address this limitation, our group developed an “umpolung” approach, utilizing hypervalent iodine compounds to create electrophilic alkyne transfer reagents such as benziodoxol­(on)­es (Bx­(X)­s) and benziodazolones (BZs). The high reactivity of EBx/X/Z reagents enables efficient alkyne transfer to various nucleophilic residues in peptides and proteins under different reaction conditions, providing a versatile tool for biomolecule modification.

In this Account, we highlight the residue-selective alkynylation and alkenylation of peptides enabled by the development of novel EBx/X/Z reagents with a focus on progress since 2021. This includes the following: (1) Selective residue modification: We have made significant progress in the residue-selective alkynylation and alkenylation of peptides and proteins. Building on our initial work with Cys-selective alkynylation, we enhanced reactivity and solubility by introducing a sulfonate group on the benziodoxolone arene core, facilitating lipophilic alkynylation in an aqueous environment. Furthermore, we developed perfluoroaryl-modified BZ reagents to achieve sequential Cys-Cys cross-linking and used them for antibody cross-linking with superior reactivity compared to that of conventional methods. Additionally, we expanded the reactivity beyond Cys to achieve Tyr-selective conjugation. All of these achievements underscored the tunability of EBx/X/Z reagents through strategic substituent modification on the iodine core. (2) Peptide stapling and macrocyclization: We designed EBx­(X) reagents featuring an additional reactive site on the alkyne moiety, enabling Cys-Cys and Cys-Lys stapling in peptides. This approach enhanced their α-helicity and potential as PPI inhibitors with improved binding affinity to the MDM2 protein. For sequences lacking Cys, we incorporated the whole EBx­(X) core onto Lys residues via an activated ester on the alkyne, forming peptide-EBx­(X) conjugates. These conjugates facilitated the formation of rigid, functional peptide macrocycles using C-terminal or Trp-selective alkynylation. The utility of these macrocyclizations was demonstrated by achieving improved binding affinity to the KEAP1 protein and by generating fluorescent cyclic peptides suitable for live-cell imaging without additional fluorophores. (3) Broadening applicability with EBx-containing amino acids: We prepared EBx amino acids compatible with both solid-phase peptide synthesis (SPPS) and solution-phase synthesis (SPS), allowing us to apply our cyclization strategies to construct a diverse library of cyclic peptides.

## Graphical abstract

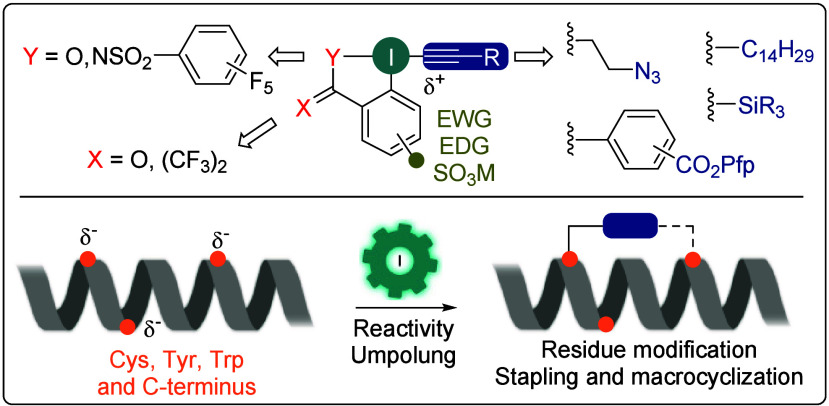

## Key References

-

Mishra, A. K. ; Tessier, R. ; Hari, D. P. ; Waser, J. Amphiphilic Iodine­(III) Reagents for the Lipophilization of Peptides in Water. Angew. Chem., Int. Ed. 2021, 60, (33), 17963–17968 10.1002/anie.202106458 PMC845693234038604.^1^ In this work, a sulfonate-containing HIR reagent enabling the lipophilization of peptides in aqueous media was developed.
-

Ceballos, J. ; Grinhagena, E. ; Sangouard, G. ; Heinis, C. ; Waser, J. Cys–Cys and Cys–Lys Stapling of Unprotected Peptides Enabled by Hypervalent Iodine Reagents. Angew. Chem., Int. Ed. 2021, 60­(16), 9022–9031 10.1002/anie.202014511 PMC804898133450121.^2^ This work introduced a bifunctional EBX reagent for rapid Cys–Cys and Cys–Lys peptide stapling, resulting in a peptide with increased α-helicity and binding affinity to MDM2.
-

Liu, X.-Y. ; Ji, X. ; Heinis, C. ; Waser, J. Peptide-Hypervalent Iodine Reagent Chimeras: Enabling Peptide Functionalization and Macrocyclization. Angew. Chem., Int. Ed. 2023, 62­(33), e202306036 10.1002/anie.202306036 37311172.^3^ This study demonstrated a proof of concept for introducing HIRs into peptides, showcasing their application in peptide functionalization and macrocyclization.
-

Liu, X.-Y. ; Cai, W. ; Ronceray, N. ; Radenovic, A. ; Fierz, B. ; Waser, J. Synthesis of Fluorescent Cyclic Peptides via Gold­(I)-Catalyzed Macrocyclization. J. Am. Chem. Soc. 2023, 145­(49), 26525–26531 10.1021/jacs.3c09261 38035635 PMC10722513.^4^ This work reported the first gold­(I)-catalyzed peptide macrocyclization proceeding via C–H functionalization, yielding cyclic peptides bearing a fluorescent linker suitable for direct live-cell imaging.
-

Liu, X.-Y. ; Mykhailenko, O. ; Faraone, A. ; Waser, J. Hypervalent Iodine Amino Acid Building Blocks for Bioorthogonal Peptide Macrocyclization. Angew. Chem., Int. Ed. 2024, 63­(33), e202404747 10.1002/anie.202404747 38807563.^5^ This study demonstrated the incorporation of HIR-containing amino acid building blocks into peptides via solid- or solution-phase synthesis, enabling diverse peptide macrocyclizations through reaction of the HIR handle.

## 1 Introduction and Context

Alkynes play a pivotal role in drug discovery,^6,7^ materials science,^8^ and chemical biology.^9^ In particular, their biorthogonal reactivity in cycloaddition reactions (“click chemistry”) is now an indispensable tool for bioconjugation.^10,11^ Furthermore, the incorporation of a rigid motif as a macrocyclic linker has emerged as a promising strategy to further enhance favorable properties of cyclic peptides, such as enhanced membrane permeability and protease stability compared to that of linear counterparts.^12,13^ Such linkers not only enforce structural constraints but also offer opportunities for further functionalization.

Traditional alkynylation approaches have largely relied on the acidic nature of the terminal alkynes. Deprotonation generates a nucleophilic acetylide, which then reacts with electrophilic sites (Scheme 1a). Typical examples include addition chemistry between alkynyl metal species and ketones or aldehydes^14^ and the Sonogashira coupling between terminal alkynes and aryl halides in the presence of Cu and Pd.^15^ Alkyne-containing peptidic macrocycles in particular have been obtained via intramolecular Sonogashira^16^ or Glaser coupling,^17^ which is often not ideal due to their reliance on toxic transition metals, harsh reaction conditions, and the need for protected peptide sequences. Moreover, rather than possessing reactive electrophilic sites, most biomolecules contain multiple nucleophilic sites such as amino, hydroxy, and thiol groups, which are better suited to engage in electrophilic alkynylation. Therefore, the development of alkyne umpolung strategies, based on the reversal of their polarity by installing electron-withdrawing groups, has opened new avenues for alkynylation (Scheme 1b).^18^ Various electrophilic alkynylation reagents, including haloalkynes,^19^ alkynyl sulfones,^20,21^ alkynyl iodoniums,^22^ and, more recently, alkynyl sulfoniums^23,24^ and ethynylbenziodoxolones (EBXs),^25–27^ have emerged for electrophilic alkynylation. However, most of the reported transformations still required harsh conditions or transition metals, leading to low biocompatibility.

**Scheme 1:**
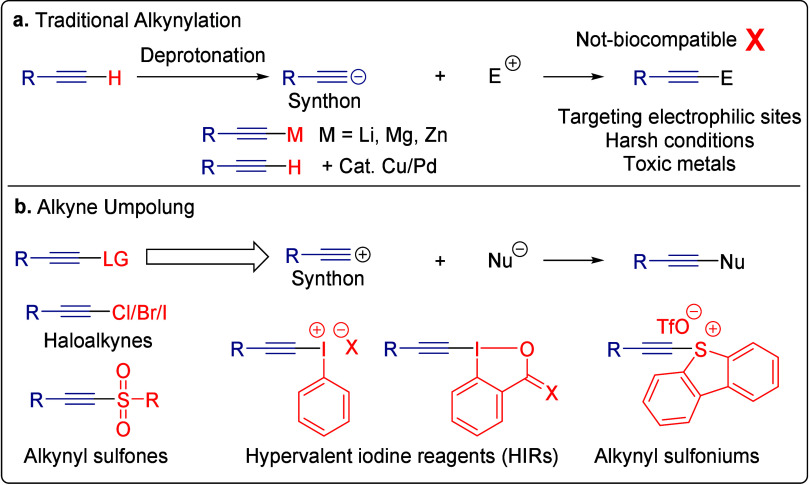
**a**. Traditional Nucleophilic Alkynylation on Small Molecules and **b**. Existing Reagents for Electrophilic Alkynylation via Alkyne Umpolung

Due to the limited availability of biocompatible methods for biomolecule alkynylation, alkynylated biomolecules widely used in medicinal chemistry and chemical biology are typically synthesized through the incorporation of alkyne-containing building blocks or via complex bioengineering approaches,^28,29^ often resulting in the use of linkers that are not essential for this function.

Our research group has long been dedicated to the development of novel transformations involving hypervalent iodine reagents (HIRs), particularly EBXs.^30–32^ Since 2013, we have been investigating selective residue modifications on peptides using HIR reagents (Scheme 2). For bioconjugation, the properties and reactivity of EBX reagents can be fine-tuned at three key sites (Scheme 2a): (1) The aryl ring, where the introduction of electron-donating or electron-withdrawing groups can significantly tune the alkynylation reactivity. (2) The heteroatom ligand on iodine, with carboxylate (EBX), bistrifluoromethylalkoxide (EBx), and amide (EBZ) displaying different reactivity and stability. (3) The alkyne motif, on which silyl, aryl, and alkyl groups containing diverse functional handles such as alkenes, alkynes, azides, alcohols, or halides can be incorporated. Furthermore, the type of substituent on the alkyne is essential to control the outcome of the reaction (addition vs alkynylation). The exceptional reactivity of cysteine (Cys) with EBXs has enabled their application not only in single-residue peptide and protein modification but also in proteomic studies (Scheme 2b).^33–37^ In these works, we showed that EBXs displayed higher reaction kinetics and Cys selectivity when compared to the “gold standard” iodoacetamide. In parallel, we have developed novel methodologies for selective residue alkynylation on tryptophan (Trp) and peptide C-termini using gold catalysis^38^ and photoredox catalysis (Scheme 2c,d).^39^

**Scheme 2:**
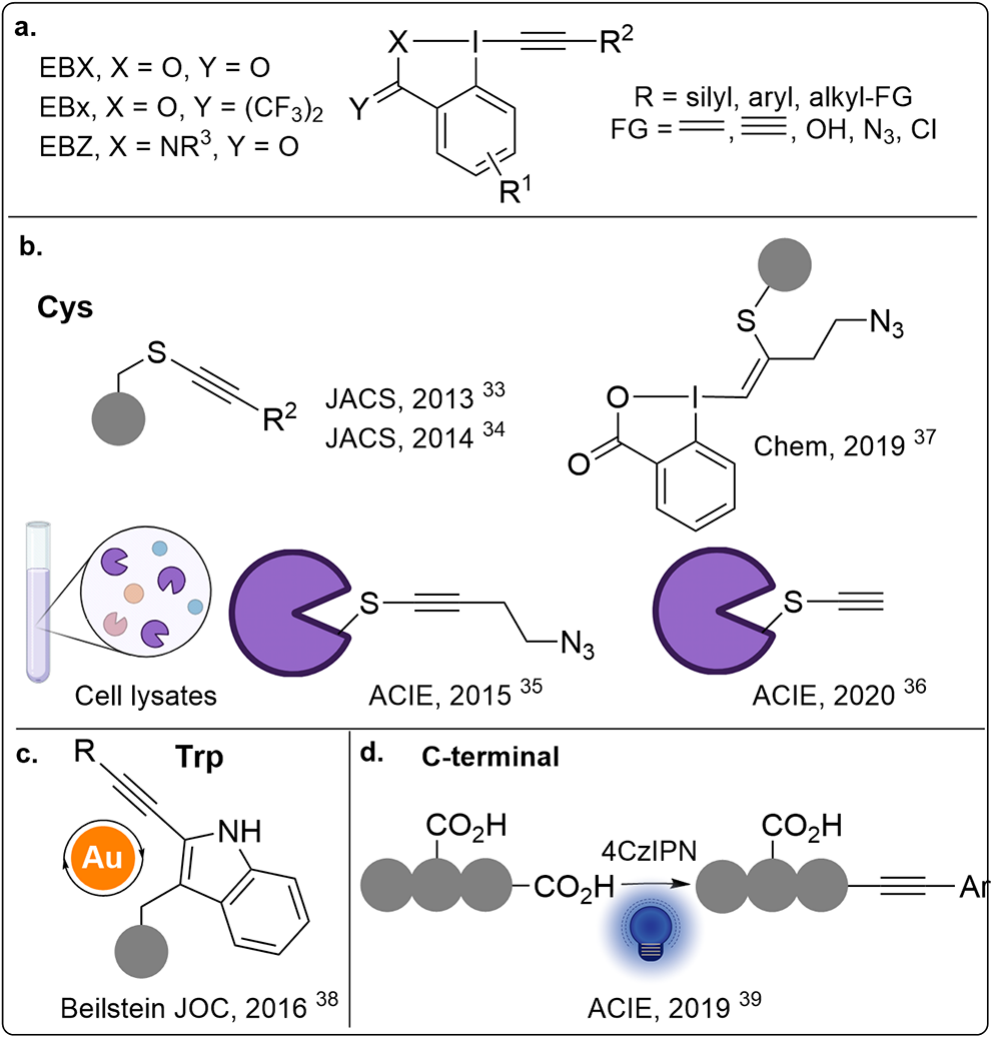
Cyclic Hypervalent Iodine Reagents: **a**. Design of and **b–d**. Early Work on Residue-Selective Peptide/Protein Modification

Many of these advances were summarized in our 2021 review on the use of hypervalent iodine reagents for biomolecule functionalization.^40^ Building on these results, our research since 2021 has focused on developing new hypervalent iodine reagents for peptide and protein modifications, with an emphasis on enhancing and expanding the scope of EBX reactivity. By strategically modifying the aryl backbone of HIRs, we transformed EBX and EBX/Z reagents into effective tools for lipid transfer and antibody rebridging. Additionally, we achieved selective tyrosine bioconjugation using the EBX reagents. We designed several bifunctional EBx­(X) reagents that enable efficient access to structurally diverse and conformationally rigid cyclic peptides featuring an alkyne cyclic linker. Important progress for site-selective modification was the introduction of hypervalent iodine reagents into peptides, via both late-stage modification and using non-natural amino acid building blocks. These alkyne-containing cyclic peptides exhibit unique properties, highlighting their strong potential in drug discovery and chemical biology applications.

## 2 Residue Modification

### 2.1 Cysteine (Cys) Modification

Cys is particularly suitable for peptide modification because of its relatively low abundance and high nucleophilicity. Since 2021, our efforts have focused on fine-tuning the reagent properties by introducing different functionalities on the phenyl ring (Scheme 3). The incorporation of a sulfonate group on the phenyl ring in reagent **1** enhanced water solubility, enabling the transfer of highly lipophilic residues (Scheme 3a).^1^ In addition to the TIPS group, long aliphatic chains (C_14_H_29_) can also be incorporated into the EBX core for lipidating peptides and proteins, which provides invaluable tools to study lipidation as a post-translational modification (PTM) of proteins.^41^ The increased lipophilicity of the modified sequences was confirmed through a comparison of reverse-phase HPLC retention times and log P determination. To mimic native lipidation PTMs, the alkyne motif was hydrolyzed under acidic conditions, forming a labile S-ester bond. The cleavage of the S-ester bond was achieved by treatment with KF (for TIPS alkynes) or hydroxylamine, effectively restoring the peptide’s original free Cys state.

**Scheme 3:**
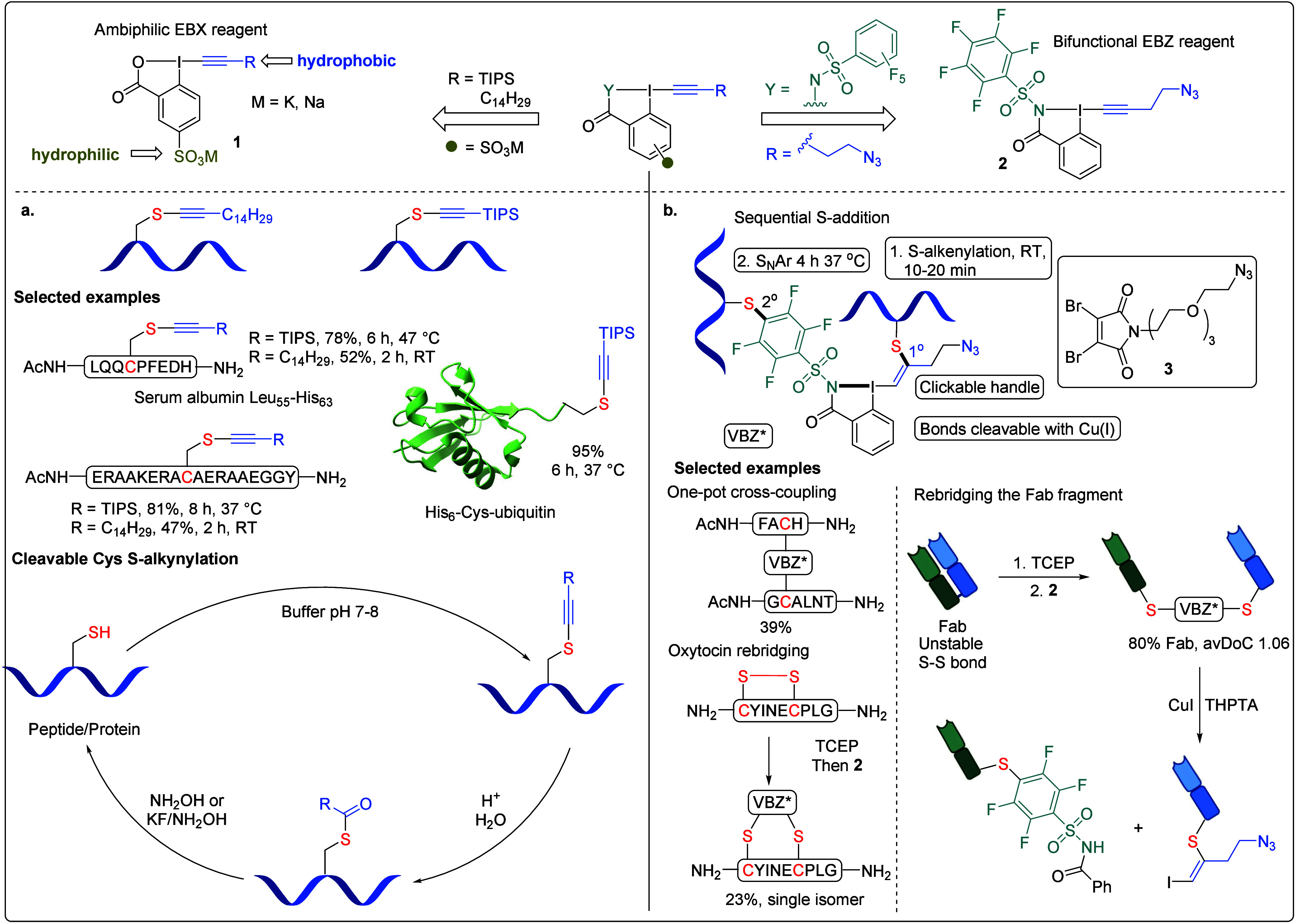
**a**. Cysteine-Selective Lipophilization and **b**. Cross-Conjugation Enabled by Ambiphilic and Bifunctional EBX/Z Reagents

Later, we synthesized ethynyl­benziodazolone (EBZ) reagent **2** featuring a pentafluoro­phenylsulfonyl motif on one side for the Cys-selective S_N_Ar reaction and an EBZ core on the other side for Cys-selective alkenylation (Scheme 3b).^42^ The two Cys reactive groups have different reactivities, with the addition of the activated alkyne occurring faster than nucleophilic aromatic substitution. This reagent enabled selective and sequential Cys modifications, allowing us to construct a unique peptide–peptide or protein–protein cross-linker with an azide handle for bio-orthogonal conjugation. The Cys addition to the EBZ core proceeded first at room temperature, yielding the *S*-alkenylation product within 30 min. In contrast, the S_N_Ar reaction between Cys and the pentafluoro­phenylsulfonyl group required elevated temperatures and extended reaction times. Notably, the resulting conjugates can be readily cleaved using CuI. Leveraging this reactivity, we rebridged the cyclic peptide oxytocin by first opening the disulfide bridge with TCEP, followed by trapping the free Cys residues with the EBZ reagent. Interestingly, this rebridging process yielded a single regioisomer. To demonstrate the utility of this reagent, we applied it to rebridge the disulfide bonds of various Fab antibody fragments,^43^ forming a stable VBZ linker with up to 84% conversion (avDoC = 1.06). The VBZ linker not only enhances structural stability but also provides an azide handle for further functionalization. In comparison, conventional dibromomaleimide reagent **3** ^44^ exhibited significantly lower rebridging efficiency, likely due to its instability under TCEP conditions. The VBZ-linked Fab can be cleaved by using CuI, further expanding its potential applications in antibody modification.

The use of disulfide exchange for the cellular uptake of oligochalcogenides is an intriguing yet underexplored topic.^45^ Developing small-molecule inhibitors to regulate this uptake process is therefore of significant interest.^46^ Together with the Matile group, we investigated the inhibition of thiol-mediated uptake using a comprehensive collection of EBX/Z reagents. (Typical structures are highlighted in Scheme 4.^47^) By leveraging high-content, high-throughput screening (HCHTS) using an FITC-ETP reporter, we disclosed that the best EBX reagent is 250 times more active than the gold standard Ellman’s reagent^48^ with a minimum inhibitory concentration (MIC) of around 2 μM.

**Scheme 4:**
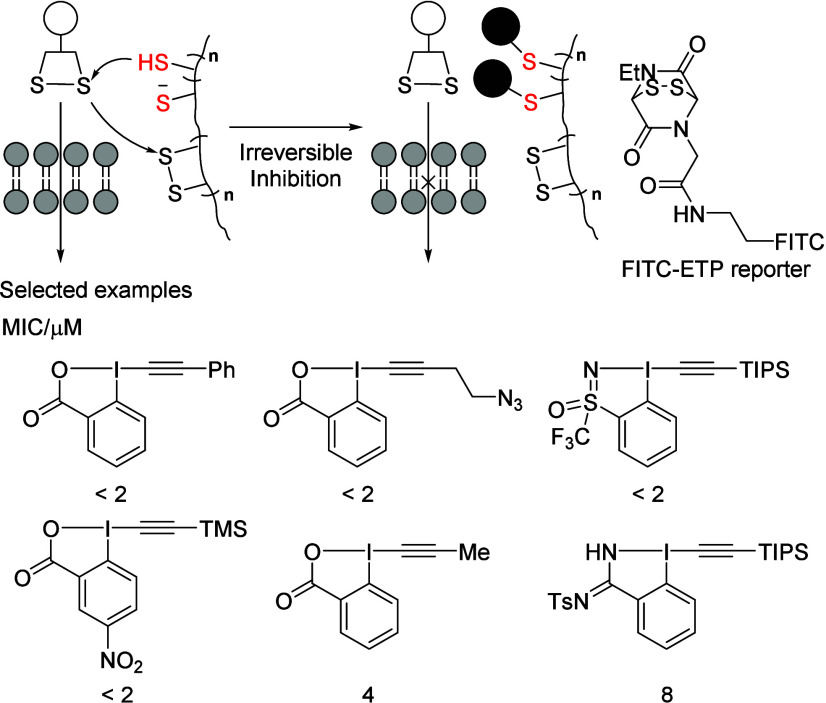
Inhibition of Thiol-Mediated Uptake by EBX/Z^a^

a Reagent MIC (minimal inhibitory concentration) needed to inhibit the thiol-mediated uptake of FITC-ETP by *ca*. 15% is given.

### 2.2 Tyrosine (Tyr) Modification

Meanwhile, we also continued our efforts toward selective residue modification with EBX reagents beyond Cys. Tyr has been regarded as another important target residue for chemical modification due to its low redox potential and flexible p*K*_a_ value based on the microenvironment of proteins.^49^ In 2022, we reported the vinylation of Tyr with the EBX reagent.^50^ In the absence of Cys, EBXs showed good reactivity toward Tyr under aqueous conditions (Scheme 5). Various EBX reagents carrying azide, alkyne, halide, hydroxyl, and fluorophore groups can be incorporated onto Tyr, leading to O-VBX conjugates (Scheme 5a). The reaction can be applied to peptides, proteins, and antibodies in moderate to excellent yields. Selectivity on proteins depends on the accessibility of the Tyr residues. The less reactive myoglobin could be functionalized only after denaturing the protein. An azide handle can be utilized for strain-promoted alkyne–azide cycloaddition (SPAAC), while the hypervalent iodine handle can be employed for bioorthogonal Suzuki cross-coupling. To demonstrate the utility of the O-VBX products, our group collaborated with the Matile group to explore its application in enhancing the thiol-mediated cellular uptake of streptavidin (Scheme 5b).^51^ First, Tyr residues on streptavidin (66 kDa) were modified with EBX, followed by Suzuki cross-coupling to introduce a lipophilic CF_3_-substituted phenyl ring, further improving cellular uptake.^52,53^ Then, the azide was utilized to install different transporters for thiol-mediated uptake (AspA, CTO). The modified streptavidin was then mixed with a TAMRA-biotin dye for uptake studies in the Hela MZ cells. Significant differences in the uptake performance were observed before and after the CuAAC reaction. With the free azide handle, punctated fluorescence indicates limited endosome escape. On the contrary, with AspA and CTO transporters 3-fold and 28-fold increases in cellular uptake were observed, respectively.

**Scheme 5:**
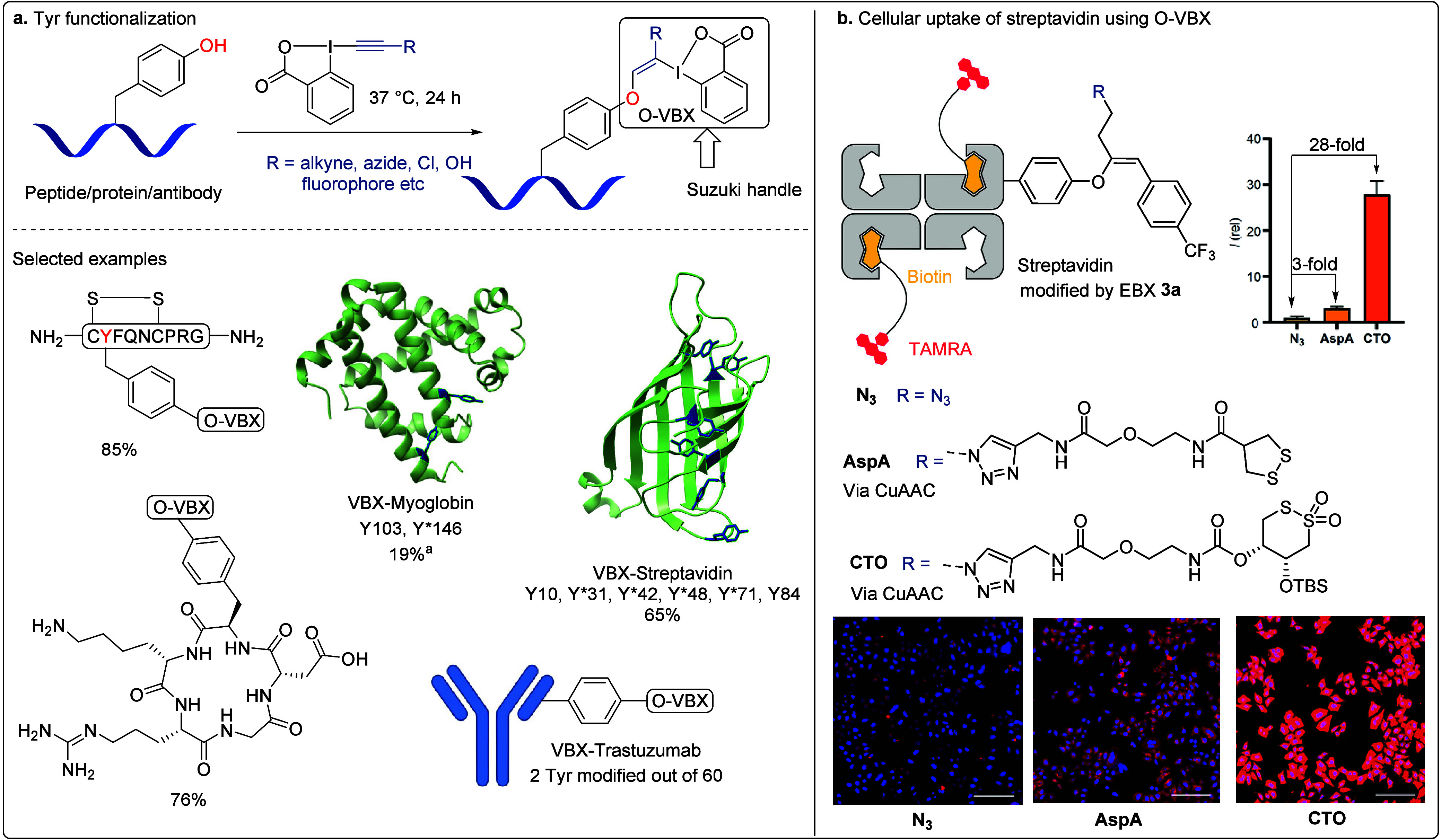
**a**. Tyrosine-Selective Modification Enabled by EBX Reagent, Modified Residues Are Indicated with an Asterisk (*) and **b**. Cellular Uptake of Streptavidin Using O-VBX in Which the Regioselectivity for Multityrosine Labeling on Proteins or Antibodies Remained Undetermined^a^

a Denatured with Tris buffer (100 mM, pH 9.0, 6.0 M GdmHCl).

## 3 EBx(X) Reagents for Peptide Stapling and Macrocyclization

To enable efficient peptide cyclization or stapling with an alkyne-based cyclic linker, we designed and synthesized a series of bifunctional EBx­(X) reagents incorporating an additional reactive group on the alkyne moiety. This reactive group must exhibit orthogonal reactivity relative to the EBX core to ensure a selective and efficient peptide modification.

### 3.1 Cysteine-Cysteine (Cys-Cys) Stapling

In 2021, we initiated our investigation into peptide cyclization enabled by EBx­(x) reagents by targeting the reaction with two cysteines first.^2^ To this end, we synthesized bis-EBx reagents featuring either an aryl or a silicon spacer (Scheme 6). These reagents exhibited excellent reactivity for stapling peptides containing adequately placed di-Cys sequences. Notably, the silicon spacer reagent showed enhanced reactivity toward simple Cys and peptide stapling relative to aryl-based counterparts. Furthermore, the stapling efficiency declined with increasing residue spacing from i/(i + 4) to i/(i + 7), except for the *para*-phenyl spacer.

**Scheme 6:**
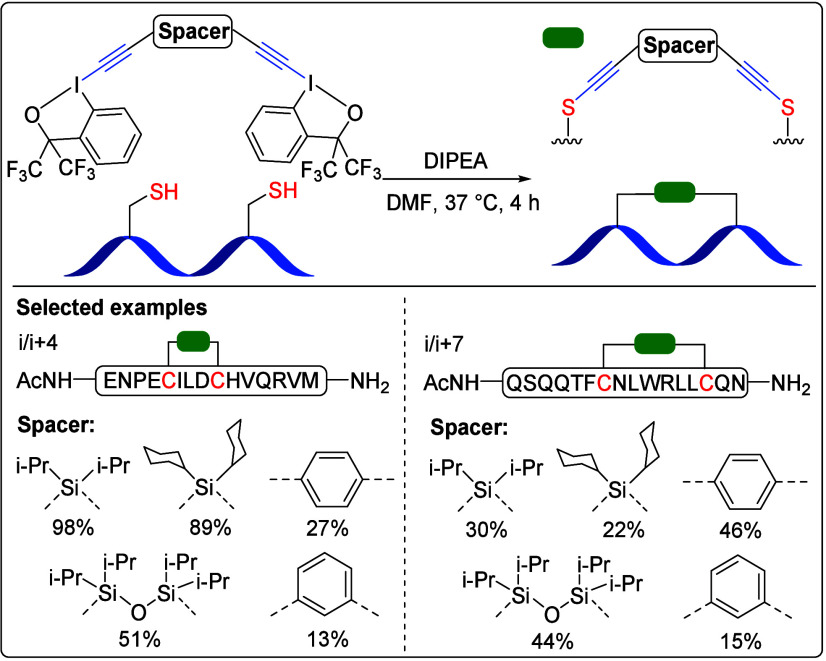
Bis-EBx Reagents for Cys-Cys Stapling

### 3.2 Cysteine-Lysine (Cys-Lys) Stapling

However, stapling with a symmetric residue is not ideal,^54^ and the structural complexity of HIRs makes them less appealing than simpler, established Cys-Cys stapling reagents. To realize nonsymmetric stapling, we prepared bifunctional EBX reagent **4** featuring an EBX core on one end and a pentafluorophenol ester on the other, targeting Cys and Lys, respectively (Scheme 7). The activated ester was positioned at the *para*, *meta*, or *ortho* positions of the phenyl ring to evaluate the influence of the substitution pattern on reactivity (Scheme 7a). Under mild basic conditions, the stapling reaction proceeded efficiently across various α-helical peptide sequences in both i/(i + 4) and i/(i + 7) manners (Scheme 7b). However, peptide staples formed with the *ortho*-substituted reagent could not be isolated due to poor product stability. The resulting S-alkyne linker was further functionalized through Ru­(II)-catalyzed azide-thioalkyne cycloaddition (RuAtAC),^55^ enabling the incorporation of diverse motifs onto the peptide cyclic linker (Scheme 7c). Notably, this method demonstrated high effectiveness in stapling α-helical peptides binding to MDM2. Circular dichroism (CD) analysis confirmed a significant enhancement in α-helicity for both i/(i + 4) and i/(i + 7) staples as well as for the cyclic peptide obtained following the RuAtAC reaction (Scheme 7d). Compared with their corresponding linear peptides, the stapled variants exhibited an approximately 12-fold improvement in binding affinity toward MDM2 (Scheme 7e). Remarkably, these staples not only demonstrated stronger binding affinity (*K*_d_ = 29 ± 4 nM) than optimized olefin metathesis staples (*K*_d_ = 55 ± 10 nM)^56^ but also avoided the generation of *E*/*Z* isomer mixtures that often complicate metathesis-based stapling. To further elucidate the reactivity order in Cys-Lys stapling, we conducted a mass spectrometry (MS) kinetic experiment (Scheme 7f). Among all possible intermediates, only the S-alkynylation product was detected, indicating that the reaction proceeds exclusively through Cys-selective alkynylation, followed by proximity-driven amidation. The kinetics of the S-alkynylation step was too fast to allow precise determination of the rate constant (70% conversion was already observed when the first data point was measured after approximately 5 s), but it could be estimated to be larger than 4 × 10^3^ M^–1^ s^–1^.

**Scheme 7:**
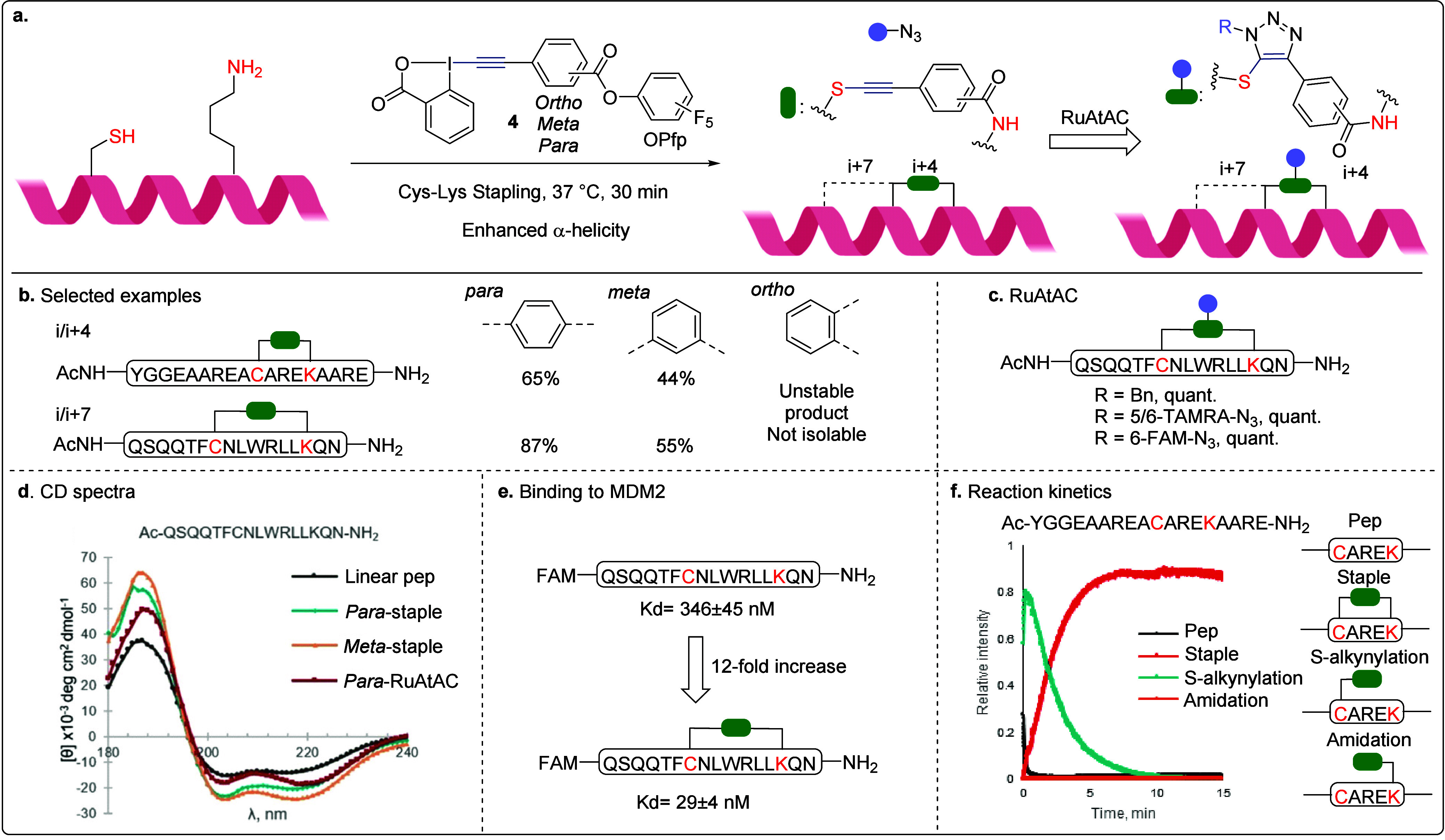
Cys-Lys Stapling of α-Helical Peptides with Bifunctional EBX Reagents: **a**. Stapling and Functionalization Protocol, **b**. Selected Examples, **c**. RuAtAC, **d**. CD Spectra, **e**. Binding to MDM2, and **f**. Reaction Kinetics

### 3.3 Peptide-EBx­(X)

Inspired by the successful application of EBX reagents in Cys-Cys and Cys-Lys stapling, we explored whether incorporating the EBx­(X) core into non-Cys-containing peptide sequences would be feasible (Scheme 8). This approach would provide an EBX handle with bio-orthogonal reactivity for peptide modifications and cyclizations. By introducing a methylene spacer between the EBX core and the activated ester in bifunctional EBX reagent **4** to give **5**, we diminished its reactivity (Scheme 8a), enabling the synthesis of a first generation of peptide-EBXs **7** through selective amidation between Lys or the N-terminus and the activated ester (Scheme 8b).^3^ The resulting peptide-EBXs maintained excellent EBX reactivity, enabling further peptide residue modifications and cross-couplings. We also developed an efficient photomediated, C-terminal-selective decarboxylative macrocyclization of peptide-EBXs promoted by the organic dye 4CzIPN (Scheme 8b). The redox potential difference between C-terminal carboxylate and side-chain carboxylate allows us to selectively react C-termini in the presence of free Asp and Glu residues. The cyclic peptides formed via intramolecular decarboxylative alkynylation exhibited highly rigid structures. An analogue of the natural product Sanguinamide A^57^ was obtained through head-to-tail decarboxylative alkynylation with excellent diastereoselectivity. The alkyne linker can be selectively reduced to a double bond with a Lindlar catalyst or to a single bond with Pd/C, giving peptides with different cyclic structures. Using reagent **5**, we cyclized several KEAP1-binding peptides, which demonstrated a significant 20-fold enhancement in binding affinity to KEAP1 compared to their linear counterparts. Notably, reducing the alkyne linker to an alkane resulted in a 5.5-fold loss of binding affinity, emphasizing the critical role of conformational rigidity in achieving high binding affinities.

**Scheme 8:**
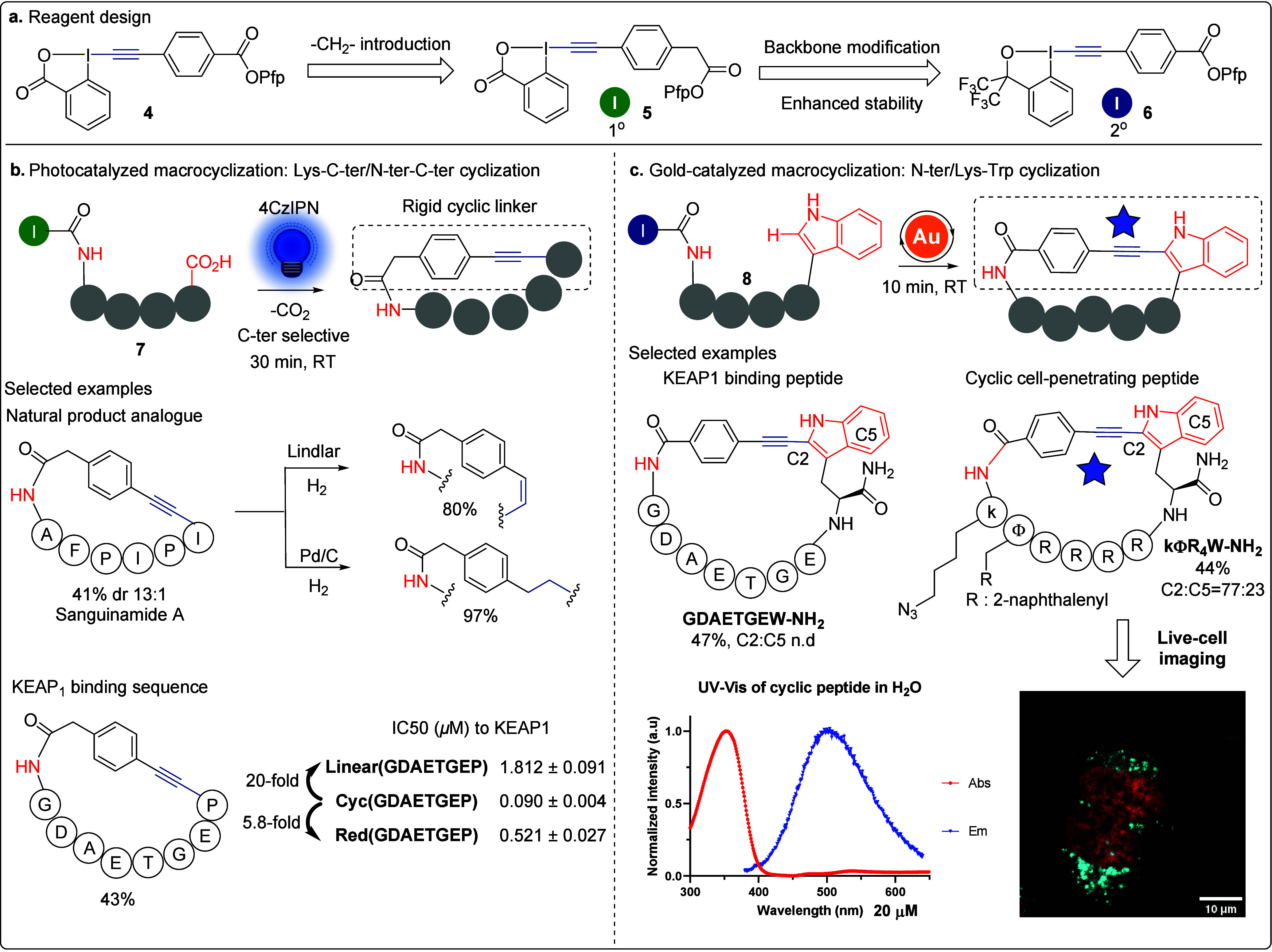
**a**. Design of New Bifunctional EBx­(X) Reagents **5** and **6** and Application of Obtained Peptide EBX Reagents **7** and **8** in **b**. Photocatalyzed and **c**. Gold-Catalyzed Peptide Macrocyclization

Although the first generation of peptide-EBXs **7** was successfully synthesized, they were obtained in low isolated yields due to the poor stability of the EBX during synthesis and purification. To address this, we developed a second generation of peptide-EBXs **8** by replacing the carbonyl backbone with a bis-CF_3_ benziodoxole backbone (EBx) (Scheme 8a,c).^4^ This modification significantly enhanced the yield of selective amidation between Lys and the activated ester and improved the stability upon purification. To further facilitate the scalable synthesis of bifunctional EBx reagents **6**, we also developed a one-pot EBx synthesis starting from inexpensive and readily available I­(I) precursors.^58^

With the second-generation peptide-EBx’s **8** in hand, we successfully extended the intermolecular gold-catalyzed Trp-selective alkynylation to an intramolecular peptide cyclization (Scheme 8c).^4^ Remarkably, this reaction represents the first example of gold catalysis employed in peptide cyclization. In most tested sequences, cyclization can be performed in 10 min with excellent chemoselectivity and functional group tolerance. The resulting cyclic peptides feature an extended aromatic system, where the indole moiety acts as an electron donor and the arene ring serves as an electron acceptor. Photophysical studies revealed significant absorption between 350 and 400 nm in DMSO or DMSO/H_2_O, along with a pronounced Stokes shift in the emission spectrum. To explore their potential for live-cell imaging, we synthesized a cell-penetrating peptide and cyclized it by using our method. Upon a 2 h incubation in HeLa cells, the cyclic peptide exhibited a strong punctated fluorescence signal, demonstrating its potential as an intrinsic fluorophore for live-cell imaging without the need for additional fluorescent labels.

### 3.4 EBx Amino Acid Building Blocks for Versatile Cyclic Peptide Synthesis

Although the selective amidation of Lys was highly efficient, introducing the EB­(X)­x core onto a specific Lys residue in the presence of multiple Lys residues required a multistep protecting group strategy. To overcome this limitation, we applied the amidation chemistry to Fmoc-protected Lys, ornithine (Orn), and diaminopropionic acid (Dap) (Scheme 9a).^5^ The amidation proceeded in quantitative yields, and the resulting crude products exhibited high purity, enabling the straightforward preparation of Fmoc-EBx amino acid building blocks **9**. The synthesis can be readily performed on a gram scale. These crude building blocks were then directly used in solid-phase peptide synthesis (SPPS), facilitating efficient peptide construction (Scheme 9b). The EBx amino acids demonstrated excellent compatibility with SPPS, enabling the efficient incorporation of the EBx core in the presence of both an N-terminus and free Lys residues. Notably, this approach allowed us to fully exploit the cyclization strategies we previously developed (Scheme 9c). We successfully constructed bicyclic peptides via a two-step process: first, a gold-catalyzed Lys-Trp stapling was followed by N-to-C amidation. Additionally, we achieved selective Lys-Lys stapling through intermolecular indole alkynylation followed by intramolecular amidation. Furthermore, we realized selective i/i+5 Lys-Cys stapling, whereas direct reaction of the peptide with bifunctional reagents gave only i/(i + 4) or i/(i + 7) stapling.^2^

**Scheme 9:**
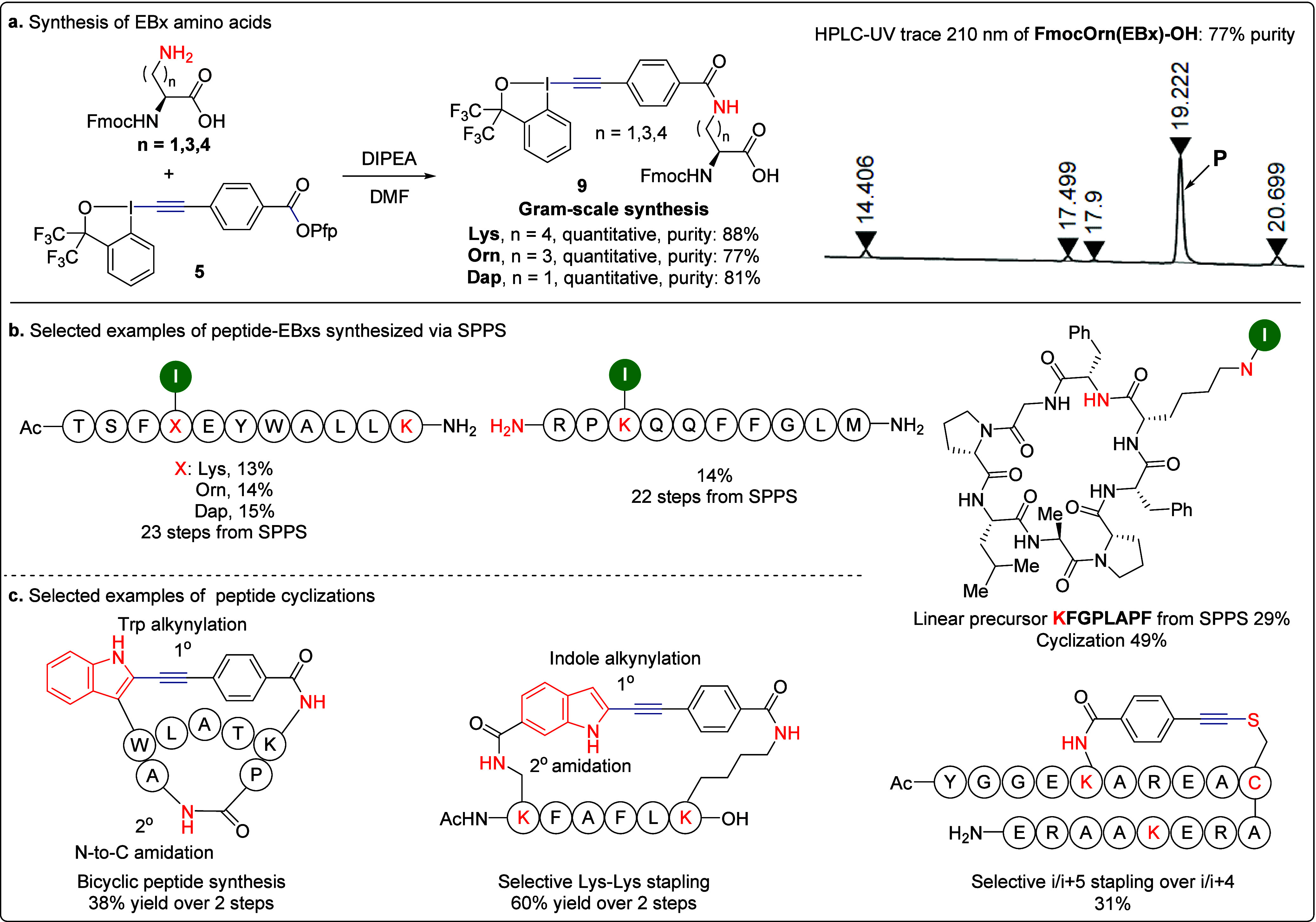
**a**. Synthesis of EBx-Amino Acids and Their Applications, **b**. Peptide-EBxs Synthesized via SPPS, and **c**. Selected Examples of Peptide Cyclizations

## 4 Conclusions and Outlook

Since the discovery of the Cys-selective alkynylation with EBX reagents,^33^ the development of new EBx­(X) reagents has greatly expanded the toolbox for biomolecule modification. The obtained functionalized biomolecules have demonstrated great potential in biological systems, serving as versatile reactive handles, stable protein–protein cross-linkers, rigid cyclic peptide linkers, and fluorophores.

Despite these advancements, the further development of selective functionalizations beyond Cys, under fully biocompatible conditions, remains both an urgent need and a significant challenge. Addressing this requires the development of new EBx­(X) reagents. We have recently developed a bicyclo[1.1.1]­pentane (BCP)-based EBX reagent whose reactivity toward a variety of nucleophiles, including N, O, and S, has been validated.^59^ This reagent shows great potential for the selective modification of amino acid residues. Moreover, its ability to introduce para-substituted benzene bioisosteres into peptides offers new opportunities for advancing peptide drug discovery. Another promising direction we are exploring is the incorporation of functional binders, such as small molecules or peptides, into the EBx­(X) core. These binders can facilitate proximity-driven residue modification by selectively guiding the reagent to specific protein binding sites.^60,61^ In this case, potentially less reactive residues can be targeted, even in the presence of highly reactive Cys.

Additionally, EBx­(X) amino acid building blocks installed on other Fmoc-amino acid building blocks will be important to increasing structural diversity. To do so, mild conditions for EBx­(X) synthesis need to be developed. Furthermore, the integration of EBx­(X) amino acids into high-throughput in vitro cyclic peptide screening platforms, such as mRNA displays,^62^ SPPS,^63^ and a DNA-encoded library (DEL),^64,65^ offers exciting opportunities. Given that several efficient EBx­(X)-mediated peptide cyclizations have already been established, incorporating these unique scaffolds into screening technologies could significantly enhance the chemical diversity of cyclic peptide libraries, which may facilitate the discovery of novel cyclic peptide binders with a high affinity and specificity for challenging protein targets.

Beyond their incorporation into peptides and proteins, the introduction of EBx­(X) into other macromolecules, such as oligonucleotides and nucleic acid-based scaffolds, would also be of great interest. This strategy would enable straightforward access to diverse peptide/protein–nucleotide conjugates featuring rigid and stable alkyne linkers, offering significant potential for applications in DNA-encoded libraries (DEL), in vivo imaging (e.g., DNA-PAINT), and targeted drug delivery.^65,66^
